# Observational study of regional aortic size referenced to body size: production of a cardiovascular magnetic resonance nomogram

**DOI:** 10.1186/1532-429X-16-9

**Published:** 2014-01-21

**Authors:** Anne E Davis, Adam J Lewandowski, Cameron J Holloway, Ntobeko AB Ntusi, Rajarshi Banerjee, Richard Nethononda, Alex Pitcher, Jane M Francis, Saul G Myerson, Paul Leeson, Tim Donovan, Stefan Neubauer, Oliver J Rider

**Affiliations:** 1 Radcliffe Department of Medicine, Division of Cardiovascular Medicine, John Radcliffe Hospital, Oxford OX3 9DU, UK; 2 Oxford Clinical Cardiovascular Research Facility, University of Oxford, Oxford, UK; 3 St. Vincent’s Hospital and the Victor Chang Cardiac Research Institute, Sydney, Australia; 4 University of Cumbria Health and Medical Sciences, Lancaster, UK

**Keywords:** Aorta, Cardiovascular magnetic resonance, Obesity, Normal Range

## Abstract

**Background:**

Cardiovascular magnetic resonance (CMR) is regarded as the gold standard for clinical assessment of the aorta, but normal dimensions are usually referenced to echocardiographic and computed tomography data and no large CMR normal reference range exists. As a result we aimed to 1) produce a normal CMR reference range of aortic diameters and 2) investigate the relationship between regional aortic size and body surface area (BSA) in a large group of healthy subjects with no vascular risk factors.

**Methods:**

447 subjects (208 male, aged 19–70 years) without identifiable cardiac risk factors (BMI range 15.7–52.6 kg/m^2^) underwent CMR at 1.5 T to determine aortic diameter at three levels: the ascending aorta (Ao) and proximal descending aorta (PDA) at the level of the pulmonary artery, and the abdominal aorta (DDA), at a level 12 cm distal to the PDA. In addition, 201 of these subjects had aortic root imaging, allowing for measurements at the level of the aortic valve annulus (AV), aortic sinuses and sinotubular junction (STJ).

**Results:**

Normal diameters (mean ±2 SD) were; AV annulus male(♂) 24.4 ± 5.4, female (♀) 21.0 ± 3.6 mm, aortic sinus♂32.4 ± 7.7, ♀27.6 ± 5.8 mm, ST-junction ♂25.0 ± 7.4, ♀21.8 ± 5.4 mm, Ao ♂26.7 ± 7.7, ♀25.5 ± 7.4 mm, PDA ♂20.6 ± 5.6, +18.9 ± 4.0 mm, DDA ♂17.6 ± 5.1, ♀16.4 ± 4.0 mm. Aortic root and thoracic aortic diameters increased at all levels measured with BSA. No gender difference was seen in the degree of dilatation with increasing BSA (p > 0.5 for all analyses).

**Conclusion:**

Across both genders, increasing body size is characterized by a modest degree of aortic dilatation, even in the absence of traditional cardiovascular risk factors.

## Background

The relationship between increasing aortic size and the risk of spontaneous rupture or dissection has been well documented [1-3]. As a result, accurate and reproducible assessment of aortic size is an essential part of a reliable clinical surveillance programme aimed at detecting progressive dilatation [4].

Although currently there are multiple modalities available for routine surveillance including computed tomography (CT), transthoracic (TTE) and transoesophageal echocardiography (TOE), cardiovascular magnetic resonance (CMR) has become the gold standard method for aortic surveillance due to its capability of accurate, reproducible imaging of the aorta in any plane, unlimited by acoustic windows, and lack of ionizing radiation. Despite this, the most widely used normal range against which other modalities, including CMR, are referenced is derived from transthoracic echocardiography datasets [5-9]. The small number of acoustic windows through which the aorta can be visualised in echocardiography limits the number of views that can be obtained, with the quality of these windows being further impaired by the presence of subcutaneous fat. Consequently, many of the imaging planes used routinely in clinical practice with CMR [10] do not have a large reference range against which to compare, reflecting the limited ability of TTE to visualise the aorta beyond the sinotubular junction (STJ).

Normal reference ranges for aortic dimensions are also available from large CT studies [11,12] but methodological differences in measurement of aortic dimension between CT and CMR, with the former measuring aortic outer wall to outer wall to derive dimensions and the latter measuring luminal diameter [13], makes direct comparison inaccurate and aortic measurements vary significantly depending on the modality used [14,15].

To date the majority of CMR studies investigating aortic dimensions have been small, and focused mainly on the aortic root [16-18] with only one prior non gender-separated CMR study investigating thoracic aortic diameter at the level of the pulmonary artery [19]. Although there are a small number of published normal measurements of the more distal thoracic aorta using computed tomography, study cohorts have either been small or have included subjects with established vascular risk factors including hypertension, which are known themselves to independently cause aortic dilatation and thus cannot provide a truly “normal” reference range [11,20]. As a response, recent American Heart Association’s guidelines for the diagnosis of thoracic disease specifically highlight the need for larger modality specific reference ranges [21,22] and as such, establishing a large normal healthy reference data for CMR aortic measurements is of clinical importance. We aimed to establish such a database of reference values for aortic diameters using cardiovascular magnetic resonance of a large diverse population of healthy volunteers, and investigate the relationship between aortic dilatation and body surface area.

## Methods

### Study cohort

447 healthy adult volunteers (male, n = 208) between the ages of 19 and 70 years without identifiable cardiovascular risk factors, were recruited to studies within the University Oxford Centre for Clinical Magnetic Resonance Research (OCMR). Subjects were grouped according to gender and World Health Organisation body mass index (BMI) categories: normal (BMI 18.5-25), overweight (BMI 25–30), obese (BMI >30), male; normal weight (55%), overweight (31%), obese (14%), female; normal weight (56%), overweight (19%), obese (25%). All subjects underwent CMR at 1.5 Tesla for the assessment of regional aortic diameter. All studies were approved by the local research ethics committee (Oxfordshire Research Ethics Committee, and informed written consent was obtained from each participant.

### Inclusion criteria

All subjects were screened for the presence of identifiable cardiovascular risk factors and were excluded if they had a history of; cardiovascular disease, cardiac chest pain, hypertension, diabetes, smoking, use of prescription medications or were pregnant or under 18 years of age (age range 18 – 80 yrs). Body mass index (BMI) in (kg/m^2^) was calculated as a simple measure of obesity using the formula weight (kg)/height(m^2^) and body surface area (BSA) in m^2^ was calculated using the Du Bois formula BSA (m^2^) = 0.20247 × Height(m)^0.725^ × Weight(kg)^0.425^.

### Blood samples

Fasting blood tests for glucose and cholesterol were taken on the day of the scanning and analysed as previously described [23].

### Blood pressure measurement

A normal blood pressure was taken as an average of three supine measures over ten minutes under 140/90 mmHg, (Model, DINAMAP 1846-SX, Critikon Corp).

### Vascular magnetic resonance

**Figure 1:**
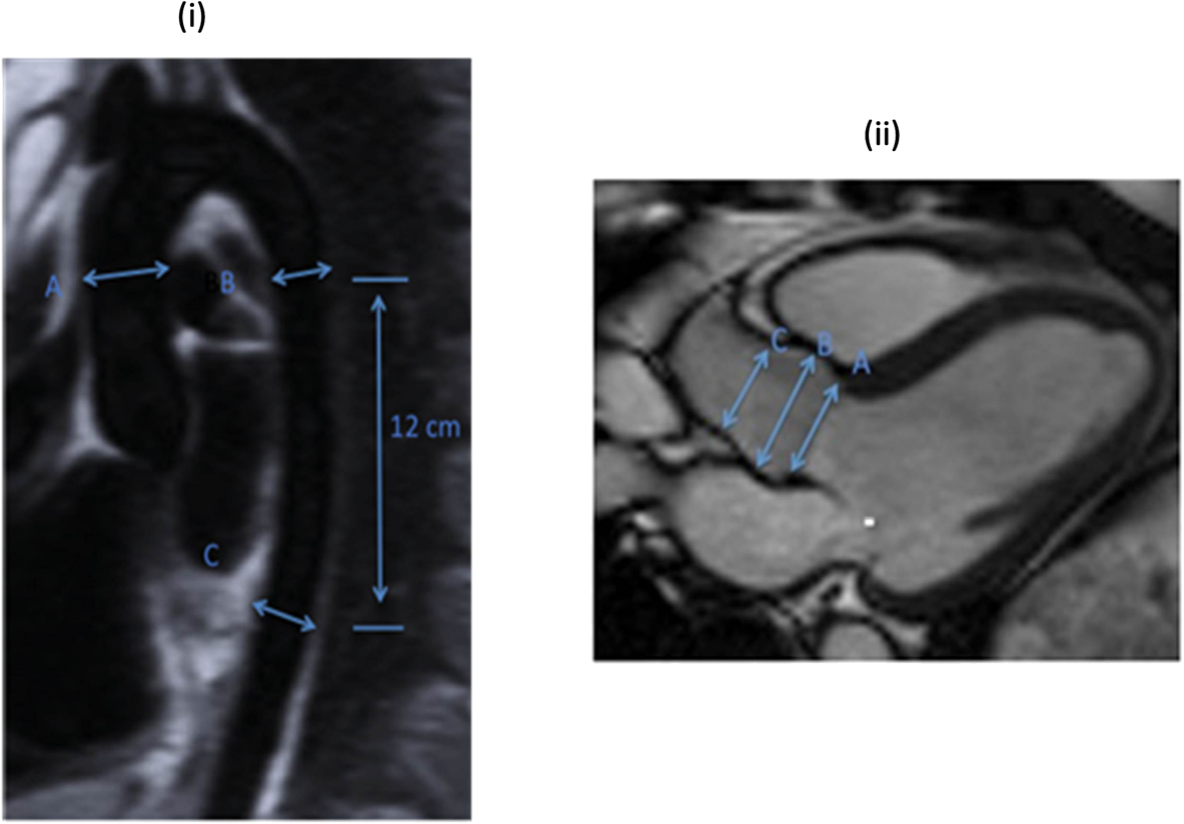
**Anatomical positions of the imaging planes used for aortic measurements. (i)** Oblique sagittal HASTE image showing the levels at which the aorta was measured using a cross-sectional SSSP sequence, (A) ascending aorta, (B) proximal descending aorta and (C) abdominal aorta. **(ii)** End-diastolic frame of an example SSFP LVOT view of the aortic root showing the levels at which the aortic root was measured (A) aortic valve annulus, (B) the widest point of the aortic sinuses and (C) the sino-tubular junction.

Imaging was performed on a 1.5 Tesla MR system (Siemens Avanto, Erlangen, Germany). All imaging was retrospectively cardiac gated with a precordial three lead ECG and acquired during end expiration breath hold. Oblique sagittal pilot, half-Fourier single shot turbo spin echo (HASTE) images were followed by steady-state free precession (SSFP) cine images with the following parameters: echo time of 1.12 ms, repetition time of 39 ms using 15 segments and 25 phases, slice thickness of 7 mm and pixel size of 2 mm × 2 mm. Cross sectional images, of the aorta were obtained orthogonal to the sagittal oblique scout at three pre-determined points; the ascending aorta (Ao) and descending aorta at the level of the pulmonary artery (PDA) together with the descending aorta 12 cm distal to the pulmonary artery (DDA) (Figure 1(i)) [13].

### Data analysis

#### Measurement of the aortic root

A sagittal oblique SSFP view of the left ventricular outflow tract (LVOT) was acquired on a smaller subset of the cohort (201 volunteers) allowing the aortic valve annulus, aortic sinus and sino-tubular junction (STJ) to be measured. Maximum diastolic diameter measurements were performed at the level of the aortic valve annulus, the aortic sinus and the sino-tubular junction (STJ) by a single reader with 8 years of CMR experience, from luminal edge to luminal edge (Figure 1(ii)). CMR can only accurately measure the lumen, as similar signal intensities between the outer wall and adjacent structures reduce delineation and thus have a detrimental effect on precision.

#### Measurement of the ascending, proximal descending and abdominal aorta

Aortic luminal area of the ascending, proximal descending and abdominal aorta was determined using in-house automated edge definition software, Matlab (version 6.5© Mathworks, Inc, Natick, MA) [24]. Diameters were then calculated from this area according to the formula

$$\mathrm{Diameter}\left(\mathrm{mm}\right)=2\times \sqrt{\frac{\mathrm{Area}\left(mm^{2}\right)}{\pi }}.$$

#### Left ventricular imaging and stroke volume analysis

To investigate any potential relationship between stroke volume and aortic dilatation which may be present in obesity, left ventricular (LV) imaging was also performed. All LV imaging was prospectively cardiac gated with a precordial three lead ECG and acquired during end expiration breathold. Images were acquired using a steady state free precession (SSFP) sequence with an echo time (TE) of 1.5 ms, a repetition time (TR) of 3.0 ms, temporal resolution 47.84 ms and a flip angle of 60° as previously described. SSFP cine sequences were used to acquire localisation images followed by a SSFP left and right ventricular short axis stack of contiguous images with a slice thickness of 7 mm and an interslice gap of 3 mm.

Image analysis for left ventricular stroke volume was performed using Siemens analytical software (Argus, version VB17, Siemens Healthcare, Erlangen, Germany). The short axis stack was analysed manually by a single cardiologist with 8 years of experience in CMR, contouring the endocardial borders from base to apex at end-diastole and end-systole as previously describe [25].

#### Statistical analysis

Participants were separated into three groups depending on their BMI; normal (BMI <25 kg/m^2^), overweight (BMI 25 – 29.9 kg/m^2^) and obese (BMI >30 kg/m^2^). All data was analysed using commercial software packages (SPSS 20; SPSS, Chicago, IL, USA and STATA, StataCorp, Texas, USA). All normally distributed results are presented as the mean ± standard deviation. All data was tested for normality using the Kolmogorov–Smirnov test. All data sets were normally distributed and analysed using one way ANOVA with Bonferoni post hoc correction. Linear regression analysis was used to assess the effect of BMI and BSA on regional aortic diameter. To compare coefficient of regression between males and females, dummy variable regression analysis was performed. An additional adjusted regression model accounting for the effects of age, height and systolic blood pressure was also performed. Values of p < 0.05 were considered as statistically significant. Normal ranges were defined as mean ± 2 standard deviations (SD).

## Results

### Anthropomorphic Data

Anthropometric data for the study groups are shown in Table 1. All subjects were normotensive at the time of scanning. Both male and female groups in each BMI category were well-matched for age, MI, diastolic blood pressure (DBP), systolic blood pressure (SBP), fasting glucose and fasting cholesterol level (all analyses p >0.05) with all values within the normal adult range. Both SBP (♂ 119 ± 11 vs. ♀ 117 ± 11 mmHg, p = 0.02) and DBP (♂ 73 ± 8 vs. ♀ 71 ± 8 mmHg, p =0.01) were statistically higher in males, but still well within the normal blood pressure range. As expected, increasing BMI was associated with an increase in both systolic and diastolic blood pressure ♂ SBP; r = 0.28, DBP; r = 0.29, ♀ SBP; r = 0.21, DBP; r = 0.18, all p <0.01).

**Table 1:** Anthropometric data separated by gender and BMI category

	**Normal weight**		**Over weight**		**Obese**	
	**(BMI <25 kg/m**^**2**^**)**		**(BMI 25 – 29.9 kg/m**^**2**^**)**		**(BMI > 30 kg/m**^**2**^**)**	
	**Male**	**Female**	**Male**	**Female**	**Male**	**Female**
**Age (years)**	36 ± 13	38 ± 12	40 ± 12	44 ± 13	46 ± 12^//^	43 ± 10
**Height (m)**	1.79 ± 0.1	1.67 ± 0.1^*^	1.78 ± 0.1	1.64 ± 0.1^*^	1.79 ± 0.1	1.64 ± 0.1^*^
**BMI (kg/m2)**	22.3 ± 1.8	21.6 ± 1.8	26.7 ± 1.2^$^	26.7 ± 1.2	33.9 ± 5.0^*//#^	36.4 ± 6.0
**BSA (m2)**	1.9 ± 0.13	1.7 ± 0.12	2.0 ± 0.13^$^	1.8 ± 0.13	2.3 ± 0.17^*//#^	2.0 ± 0.16
**Weight (kg)**	72 ± 8	60 ± 7^*$^	86 ± 8^$^	72 ± 7^*^	109 ± 17^*//#^	98 ± 15
**Glucose (mmol/L)**	4.8 ± 0.4	4.6 ± 0.4	4.9 ± 0.5	4.6 ± 0.6	5.1 ± 0.6	4.9 ± 0.5
**Cholesterol (mmol/L)**	4.4 ± 1.0	4.8 ± 0.8	4.9 ± 0.9	4.7 ± 0.8	4.9 ± 0.9	5.0 ± 0.8
**Systolic blood pressure (mmHg)**	118 ± 11	115 ± 11	120 ± 11	120 ± 12	126 ± 9^*//^	119 ± 13
**Diastolic blood pressure (mmHg)**	72 ± 8	70 ± 7	73 ± 8^#^	72 ± 10	77 ± 7^*^	74 ± 8

^*^p < 0.05 male vs female.

^#^p < 0.05 overweight vs obese.

^$^p < 0.05 normal weight vs overweight.

^//^p < 0.05 normal weight vs obese.

### Gender-specific normal aortic dimensions

**Figure 2:**
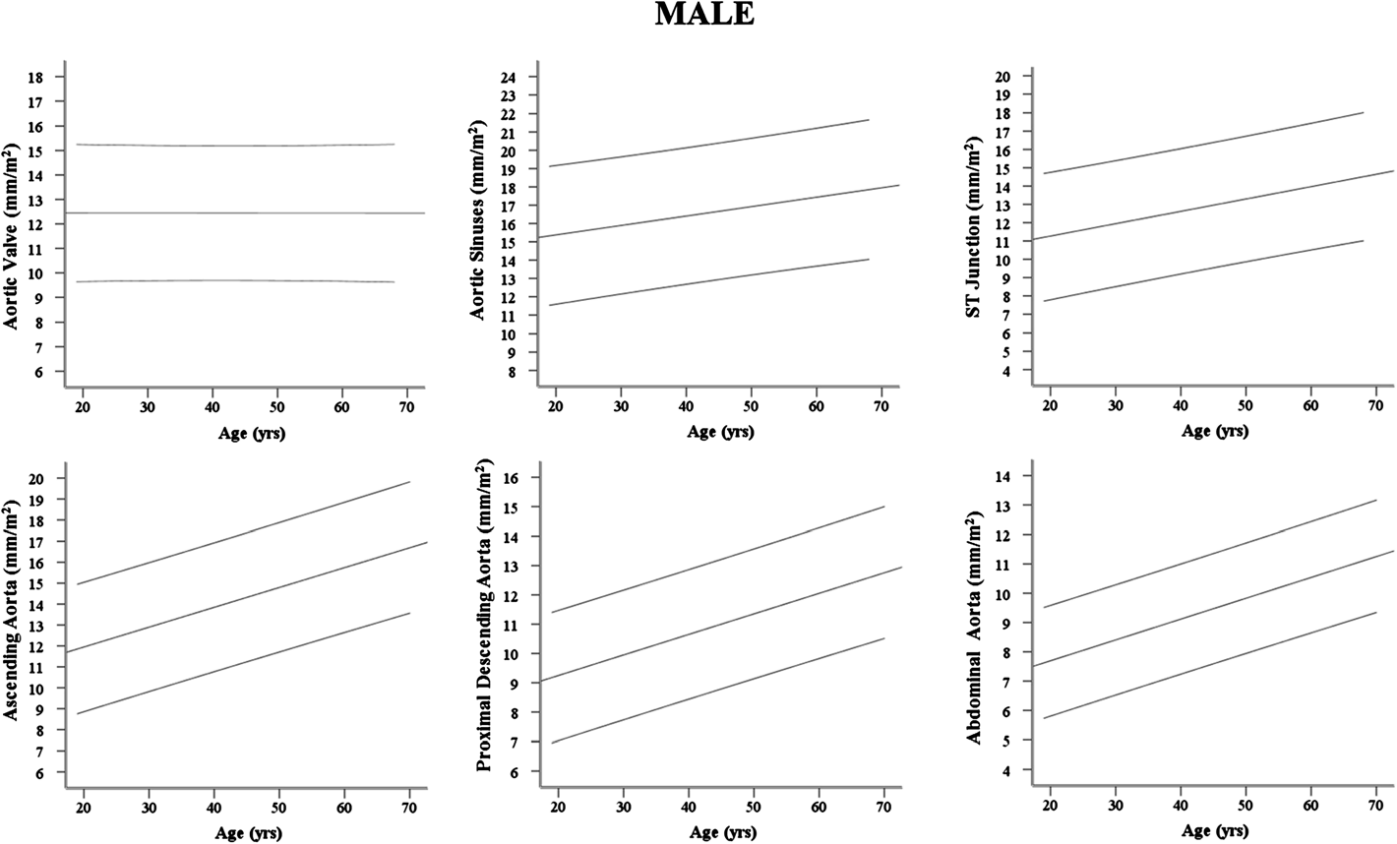
**Male normal ranges for regional aortic diameter (mm/m**^**2**^**) plotted against age.**

**Figure 3:**
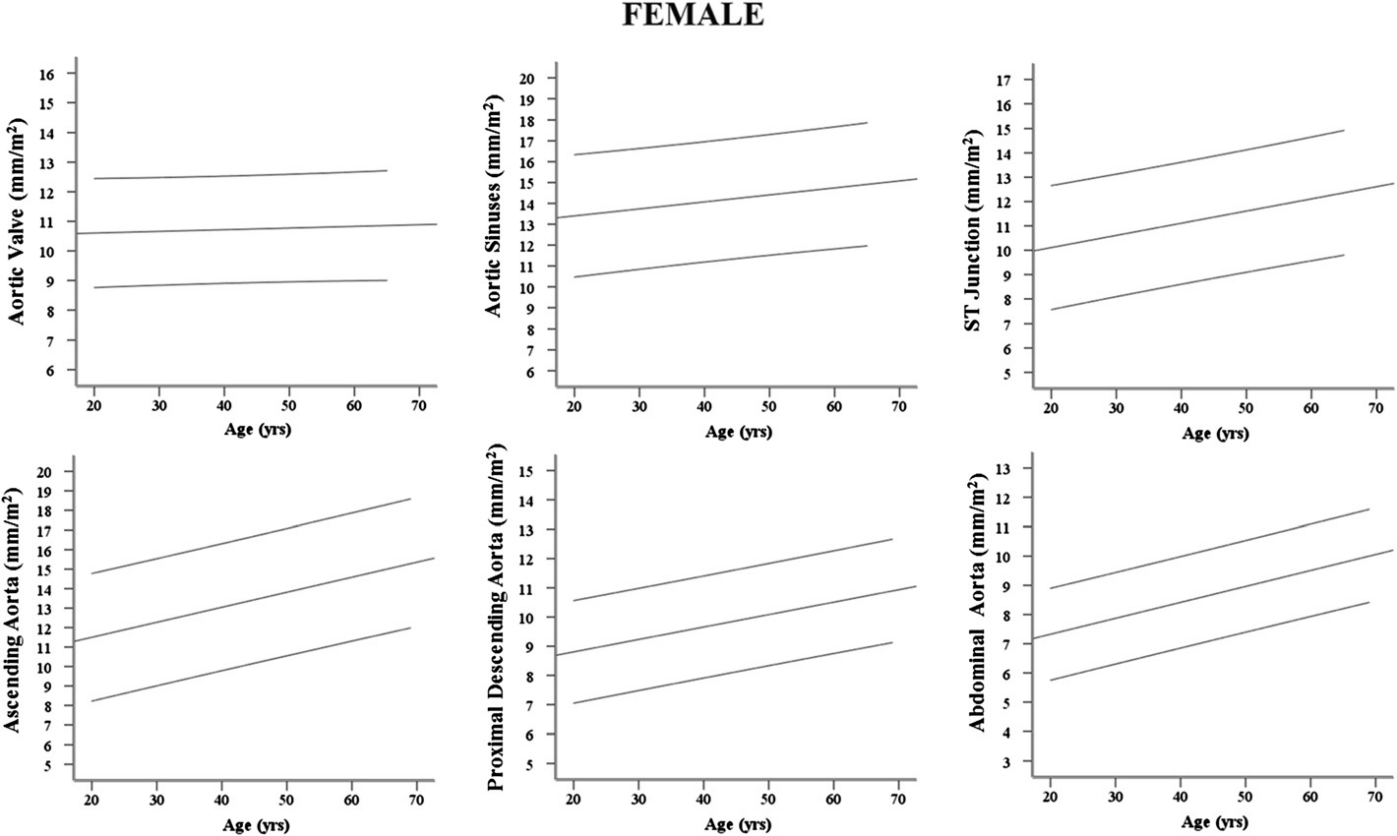
**Female normal ranges for regional aortic diameter (mm/m**^**2**^**) plotted against age.**

On grouped analysis, the mean and normal range (calculated as mean ± 2 SD) for each aortic plane across the whole population were calculated independently for male and females as follows; AV annulus male (♂) 24.4 ± 5.4, female (♀) 21.0 ± 3.6 mm, aortic sinus ♂32.4 ± 7.7, ♀27.6 ±5.8 mm, STJ ♂25.0 ± 7.4, ♀21.8 ± 5.4 mm, Ao ♂26.7 ± 7.7, ♀25.5 ± 7.4 mm, PDA ♂20.6 ± 5.6, ♀18.9 ± 4.0 mm, DDA ♂17.6 ± 5.1, ♀16.4 ± 4.0 mm. A graphical representation of the gender-specific normal range data for regional aortic diameter indexed to BSA according to age is presented as a nomogram in Figure 2 (Male) and Figure 3 (Female), while gender-specific normal range data for regional aortic diameter according to BMI group is presented in Table 2.

**Table 2:** Gender specific effects of obesity on regional aortic diameter - data presented as mean with normal range (+/- 2SD)

	**Male**			**Female**		
**Aortic diameter (mm)**	**Normal weight**	**Overweight**	**Obese**	**Normal weight**	**Overweight**	**Obese**
**Aortic valve annulus**	23.9 (18.6–29.2)	24.3 (18.9–29.7)	25.6 (20.4–30.8)	20.6 (17.4–23.8)	21.7 (18.4–25.0)	21.5 (17.2–25.8)
**Aortic sinuses**	31.9 (24.3–39.5)	32.8 (25.2–40.4)	33.3 (24.3–42.3)	27.5 (21.9–33.1)	28.0 (21.8–34.2)	27.5 (21.3–33.7)
**Sino–tubular junction**	24.4 (18.2–30.6)	25.7 (16.7–34.7)	26.2 (18.9–33.5)	21.6 (16.6–26.6)	22.3 (17.0–27.6)	22.1 (15.9–28.3)
**Ascending aorta**	26.0 (18.7–33.3)	27.4 (18.9–35.9)	28.5 (23.1–33.9)	24.7 (17.8–31.6)	26.5 (19.3–33.7)	26.6 (18.8–34.4)
**Proximal descending aorta**	20.1 (14.7–25.5)	20.9 (15.6–26.2)	22.2 (16.3–28.1)	18.5 (14.6–22.4)	19.2 (14.8–23.6)	19.6 (16.5–23.2)
**Abdominal aorta**	17.1 (12.0–22.2)	17.9 (12.8–23.0)	18.8 (14.4–23.2)	16.0 (12.1–19.9)	16.3 (12.3–20.3)	17.4 (13.9–20.9)

### Gender-specific effects of Age, blood pressure and height on regional aortic diameter

With the exception of the aortic valve annulus, which was not affected by increasing age (p > 0.44 for males and females), all other aortic measurements increased with increasing age (Tables 3 and 2, Figures 2 and 3). However, increasing age, in the absence of hypertension and other vascular risk factors, was associated with only a small change in aortic size (♂;+1.0 -1.9 mm, ♀; +0.7 – 1.5 mm per decade of increasing age). Whereas the aortic sinuses, ST junction and ascending aorta showed no gender difference in the degree of dilatation with increasing age, p > 0.16 for all analyses (Table 4), males showed a greater degree of dilatation of the proximal descending (♂; +1.4 mm vs ♀; +0.8 mm per decade ↑age, p <0.001) and abdominal aorta (♂;+1.4 mm vs ♀; +1.1 mm per decade ↑age , p <0.02, Figure 2).

**Table 3:** Correlations of regional aortic diameter

			***Male***			
	***Aortic valve annulus***	***Aortic sinuses***	***ST junction***	***Ascending aorta***	***Proximal descending aorta***	***Abdominal aorta***
	***r , p***	***r , p***	***r , p***	***r , p***	***r , p***	***r , p***
**Body mass index (kg/m**^**2**^**)**	0.30 , <0.01	0.20 , 0.06	0.27 , <0.01	0.25 , <0.001	0.27 , <0.001	0.25 , <0.001
**Body surface area (m2)**	0.37 , <0.001	0.28 , <0.01	0.37 , <0.001	0.35 , <0.001	0.35 , <0.001	0.27 , <0.001
**Age (yrs)**	-0.001 , 0.99	0.32 , 0.001	0.44 , <0.001	0.60 , <0.001	0.63 , <0.001	0.69 , <0.001
**SBP (mmHg)**	0.038 , 0.72	0.16 , 0.13	0.11 , 0.30	0.35 , <0.001	0.40 , <0.001	0.42 , <0.001
**DBP (mmHg)**	0.007 , 0.94	0.23 , 0.03	0.23 , 0.03	0.22 , 0.002	0.27 , <0.001	0.30 , <0.001
**Height (cm)**	0.23 , 0.03	0.20 , 0.06	0.23 , 0.02	0.23 , 0.001	0.20 , 0.003	0.10 , 0.14
**Stroke volume (ml)**	0.38 , <0.001	0.23 , 0.03	0.11 , 0.26	0.35 , < 0.001	0.31 , <0.001	0.30 , <0.001
			***Female***			
	***Aortic valve annulus***	***Aortic sinuses***	***ST junction***	***Ascending aorta***	***Proximal descending aorta***	***Abdominal aorta***
	***r , p***	***r , p***	***r , p***	***r , p***	***r , p***	***r , p***
**Body mass index (kg/m**^**2**^**)**	0.36 , <0.001	0.11 , 0.26	0.23 , <0.001	0.31 , <0.001	0.27 , <0.001	0.296 , <0.001
**Body surface area (m2)**	0.54 , <0.001	0.27 , <0.001	0.31 , 0.001	0.29 , <0.001	0.28 , <0.001	0.29 , <0.001
**Age (yrs)**	0.07 , 0.47	0.25 , 0.01	0.40 , <0.001	0.49 , <0.001	0.50 , <0.001	0.64 , <0.001
**SBP (mmHg)**	0.11 , 0.28	0.12 , 0.23	0.26 , 0.001	0.31 , <0.001	0.32 , <0.001	0.36 , <0.001
**DPB (mmHg)**	0.10 , 0.30	0.25 , 0.01	0.26 , 0.01	0.17 , 0.01	0.15 , 0.02	0.20 , 0.02
**Height (cm)**	0.27 , 0.01	0.27 , 0.01	0.13 , 0.18	-0.15 , 0.82	0.19 , 0.78	0.003 , 0.97
**Stroke volume (ml)**	0.43 , <0.001	0.16 , 0.10	0.23 , 0.02	0.19 , 0.004	0.18 , 0.006	0.20 , 0.02

**Table 4:** Gender specific effects of increasing BMI, BSA and age on regional aortic diameter

		**BMI**				**BSA**				**Age**			
		**R**	**β**	**p**	**p ♂**	**R**	**β**	**p**	**p ♂**	**R**	**β**	**p**	**p ♂**
					**vs ♀**				**vs ♀**				**vs ♀**
**Aortic annulus**	♂	0.3	0.17	<0.01	0.15	0.37	5.6	<0.001	0.63	0.07	0	>0.99	-
	♀	0.36	0.09	<0.001		0.54	4.9	<0.001		0.01	0.01	0.44	
**Sinus of valsalva**	♂	0.2	0.16	0.06	0.18	0.28	6	<0.01	0.43	0.32	0.1	<0.01	0.37
	♀	0.11	0.04	0.26		0.27	4	<0.01		0.25	0.07	<0.01	
**Sino tubular junction**	♂	0.27	0.21	<0.01	0.12	0.37	7.6	<0.001	0.15	0.44	0.13	<0.001	0.34
	♀	0.26	0.09	<0.02		0.31	4.3	<0.01		0.4	0.1	<0.01	
**Ascending aorta**	♂	0.25	0.22	<0.001	0.49	0.35	7.6	<0.001	0.28	0.61	0.19	<0.001	0.16
	♀	0.31	0.17	<0.001		0.29	5.6	<0.001		0.49	0.15	<0.001	
**Proximal descending aorta**	♂	0.27	0.17	<0.001	0.03	0.35	5.5	<0.001	0.03	0.63	0.14	<0.001	<0.001
	♀	0.27	0.08	<0.001		0.28	2.8	<0.001		0.5	0.08	<0.001	
**Distal descending aorta**	♂	0.25	0.14	<0.001	0.17	0.27	3.8	<0.001	0.51	0.67	0.14	<0.001	<0.02
	♀	0.29	0.09	<0.001		0.29	3.1	<0.001		0.64	0.11	<0.001	

### Gender-specific effects of increasing BMI and BSA on aortic size

In both males and females on linear regression analyses, BMI and BSA were positively correlated with all aortic diameters with the exception of the aortic sinuses, where BMI was not related to diameter (both genders; p > 0.06, Table 1). Interestingly, the degree of dilatation with increasing obesity was small, (♂;+1.4 – 2.1 mm, ♀; +0.8 – 1.7 mm per 10 kg/m^2^ increase in BMI). In general, the degree of aortic dilatation with increasing BMI and BSA was not significantly different between men and women at all levels measured (p > 0.10 for all analyses except for the level of the PDA, Table 4, Figures 2 and 3). To account for the effect of age, height and SBP, all known to increase aortic size and positively correlated with diameter in this study, an adjusted model was performed. This showed that after adjusting for age, height and SBP, the positive relationship between aortic diameters and both BMI and BSA remained unchanged (data not shown).

Again, with the exception of the aortic sinuses, all regional aortic diameter measures were positively correlated with both BMI and BSA (data not shown), with the degree of dilatation remaining small (♂;+0.5 – 1.2 mm, ♀; +0.7 – 1.2 mm per 10 kg/m^2^ increase in BMI). Again, no gender differences in the degree of dilatation with increasing BMI and BSA was seen when adjusting for systolic blood pressure, age and height.

In males and females, the largest increase in aortic size with increasing body size was at the level of the ascending aorta (♂ + 7.6, ♀ + 5.6 mm per m^2^ BSA increase, both p <0.01) with a smaller effect seen in the more distal proximal descending (♂ + 5.5, ♀ + 2.8 mm per m^2^ BSA increase, both p <0.01) and abdominal aorta (♂ + 3.8, ♀ + 3.1 mm per m^2^ BSA increase, both p <0.01). Interestingly, the effect of stroke volume increase on aortic diameter increase was also greatest in the ascending aorta (♂ + 6.5, ♀ + 3.6 mm per 10 ml increase in LV stroke volume, both p <0.01) when compared to the abdominal aorta (♂ + 3.7, ♀ + 2.4 mm per 10 ml increase in LV stroke volume, both p <0.01). Importantly, as expected, obesity was positively correlated with stroke volume (r = 0.21, p <0.001). This suggests that increased stroke volume may be accounting for at least some of the increase in proximal aortic diameters seen.

### Intra and inter observer variability

For aortic root analysis, intra-observer variability of ±0.7% (0.19 mm) was determined by blinded, repeat analysis of 15 scans (45 measurements, 5% of the study cohort) undertaken 1 week following the initial analysis. Inter observer variability was performed on the same cohort, the inter-observer variability was ±1.34% (0.36 mm). In the other regions aortic cross sectional diameter was calculated using a semi-automated in house software program within Matlab 6.5©, which results in a highly reproducible assessment of area, and more reproducible than manual contouring techniques, the coefficient of variance for the automated image analysis technique is 0.58% [26].

## Discussion

Although CMR is considered to be the gold standard for serial anatomical imaging of the aorta, a large CMR based reference range of regional aortic measurements is not currently available and many centres use echocardiography based reference ranges. Our study provides a gender specific nomogram indexed to BSA according to age using CMR. We report a modest increase in aortic size with both increased BSA and age across males and females.

### The effect of BSA on aortic diameter

Both cardiac output and total blood volume are elevated with increased BSA, and studies have shown that these circulatory changes result in left and right ventricular hypertrophy and cavity dilatation [3,27]. As a result of this increased stroke volume, it might be expected that obesity and increased BSA would be associated with an increase in aortic size, and previous studies using both CMR and echocardiography have confirmed that aortic root size is larger in obese when compared to normal weight subjects [10,28]. However, these studies have not excluded patients with hypertension and other cardiovascular risk factors that can be present in obesity and known to be independently linked to increased aortic size. As such the effect of increased BSA *per se* is not well understood. Using a large population of subjects, free from hypertension and other vascular risk factors, this study not only shows that increasing BSA and BMI, without comorbidity, is associated with increasing aortic size but also, interestingly, that the degree of dilatation in the absence of hypertension is modest, between 1.4 – 2.1 mm (males) and 0.8 – 1.7 mm (females) per 10 kg/m^2^ BMI point increase. This would suggest that if significant aortic dilatation is present in obesity, is unlikely to be attributable to obesity alone, and a second pathological process should be sought. As expected, males had larger aortic measurements than females. However, there was no gender difference in the degree of dilatation with increasing BMI or BSA.

Interestingly, the effect of increasing BSA on aortic size seems to be most pronounced in the aortic root and ascending aorta, with a smaller effect seen distally. This pattern is the mirror image of increased aortic stiffness seen in obesity, which is more pronounced in the abdominal aorta than the ascending aorta [29], and may be explained by the proximal aorta being more directly exposed to the increased stroke volume accompanying larger fat mass and therefore showing the largest change in size, in line with its 'Windkessel’ function. In support of this, not only were aortic diameters positively correlated with left ventricular stroke volume, but also the largest effect was seen in the ascending aorta. In contrast to this, volumetric changes would not be expected alter aortic elastic function, and the changes in abdominal aortic stiffness in obesity have been proposed to be related to elevated levels of adipokines and free fatty acids [30,31].

### Effect of Age on aortic size

In line with other published data, [32] increasing age was associated with modest increase in aortic size. This may be explained by the age-related increases in collagen synthesis that occur with advancing age, potentially leading to dilatation [33]. Whereas proximal sections (up to and including ascending aorta) show no gender difference in rate of dilatation with advancing age, this study has, however, shown that the more distal aortic regions (proximal descending and abdominal aorta) have significant gender differences in the degree of dilatation with advancing age, with males exhibiting up to a 43% greater dilatation than females. The reasons for this gender difference is unknown, but given the higher rate of abdominal aneurysm formation in men [34] this is an interesting finding, worthy of further study.

### Comparison with other modalities and CMR studies

Aortic sinus measurements made on this study were similar to a previous echocardiography study by Wolak et al., of 1207 subjects over 15 yrs of age [11]. Our measurements were between 3% (male) and 6% of the echo based measurements. Differences are likely to reflect a difference in measurement technique (leading edge convention in echo) and differences in cohorts, with risk factors not being excluded in the vast majority of previous studies. When compared to a large non-contrast enhanced gated CT study by Devereaux et al. of 4387 subjects, measuring ascending and descending aortic diameters at the level of the pulmonary artery [9] our results show an aortic diameter that is consistently lower than that recorded in this study (ascending aorta by 42% for both males and females, descending aorta by 18% in males and 15% in females). Possible explanations for this include differences in measurement techniques (outer edge to outer edge used in the CT study versus Inner edge to inner edge in this MR study) and the comparison of differing cohorts. Indeed, comparison with this large CT study is limited given the fact that it enrolled subjects with established cardiovascular risk factors including hypertension (27%), smoking (40%), diabetes (5%) and dyslipidaemia (36%) all which are known to exert independent effects on aortic size and/or function. Aortic root measurements in this study are very similar to those recorded in a recent study by Burman et al. [13]. This is expected given the fact that both utilise SSFP CMR at 1.5 T to measure aortic root diameter in a group of subjects with little or no cardiovascular risk factors (Burman et al. including subjects with systolic blood pressure up to 150 mmHg).

### Study limitations

This study is cross-sectional in design and does not examine changes over time related to age and increasing BSA, and is only a comparison of differing cohorts. BMI is a simple measure that is strongly correlated with sophisticated measures of obesity, but can be a misleading measure of obesity in subjects with high levels of muscle mass with normal adipose tissue levels. We are confident that in this sedentary population the elevated BMI values were not driven by elevated muscle mass. To confirm this, on separate analysis, (not shown) increased BMI was directly correlated to increased fat mass not lean mass on bioimpedance analysis, suggesting that the elevated BMI measures seen in this study were driven primarily by fat mass, not lean muscle mass.

Aortic root dimensions were measured in a single longitudinal plane, and further measurements using a second, 'coronal’ LVOT, view or short-axis cross-sectional planes may allow for a more accurate assessment of diameter. Standard long axis planes are routinely acquired in CMR studies however, and would be applicable to widespread clinical practice.

Aortic dimensions were measured at six locations along the aorta. Although five of these positions are in standardised anatomical positions, the abdominal images are acquired at an arbitrary point 12 cm distal to the pulmonary, and not located at an anatomical landmark. Despite this, the location of this slice does allow repeat clinical studies to be performed in subjects.

## Conclusion

We present a normal CMR reference range for BSA normalized aortic diameters, correlated to age at six points along the aorta. Increasing BSA is accompanied by a modest increase in aortic size in healthy subjects without identifiable vascular risk factors, even after adjusting for the effects of age, blood pressure and height. Although men had larger aortic diameters at all levels measured, there were no significant gender differences in the degree of aortic dilatation with increasing body surface area.

## Competing interests

The authors declare that they have no competing interests.

## Authors’ contributions

OR, SN, PL, SM AD made substantial contributions to conception and design of the study. AL, CJH, NN, RB, RN, AP, JMF, OR acquired the CMR data. AD, OR analysed and interpreted the data. OR, AD, NN drafted the manuscript. TD, OR, AD, SN, PL revised the manuscript critically for important intellectual content. All authors read and approved the final manuscript.
